# Overexpression of Karyopherin 2 in Human Ovarian Malignant Germ Cell Tumor Correlates with Poor Prognosis

**DOI:** 10.1371/journal.pone.0042992

**Published:** 2012-09-04

**Authors:** Li He, Hui Ding, Jian-Hua Wang, Yun Zhou, Li Li, Yan-Hong Yu, Long Huang, Wei-Hua Jia, Musheng Zeng, Jing-Ping Yun, Rong-Zhen Luo, Min Zheng

**Affiliations:** 1 State Key Laboratory of Oncology in Southern China, Cancer Center, Sun Yat-sen University, Guangzhou, Guangdong, People's Republic of China; 2 Department of Gynecology, Cancer Center, Sun Yat-sen University, Guangzhou, Guangdong, People's Republic of China; 3 Department of Pathology, Cancer Center, Sun Yat-sen University, Guangzhou, Guangdong, People's Republic of China; 4 Department of Chest, Second People's Hospital of Guangdong Province, Guangzhou, Guangdong, People's Republic of China; 5 Department of Gynecology, Huang-pu District Hospital, First Affiliated Hospital of Sun Yat-sen University, Guangzhou, Guangdong, People's Republic of China; 6 Department of Obstetrics and Gynecology, Nanfang Hospital, Southern Medical University, Guangzhou, Guangdong, People's Republic of China; H. Lee Moffitt Cancer Center & Research Institute, United States of America

## Abstract

**Background:**

The aim of this study was to identify a biomarker useful in the diagnosis and therapy of ovarian malignant germ cell tumor (OMGCT).

**Methods:**

The karyopherin 2 (KPNA2) expression in OMGCT and normal ovarian tissue was determined by standard gene microarray assays, and further validated by a quantitative RT-PCR and immunohistochemistry. The correlation between KPNA2 expression in OMGCT and certain clinicopathological features were analyzed. Expression of SALL4, a stem cell marker, was also examined in comparison with KPNA2.

**Results:**

KPNA2 was found to be over-expressed by approximately eight-fold in yolk sac tumors and immature teratomas compared to normal ovarian tissue by microarray assays. Overexpression was detected in yolk sac tumors, immature teratomas, dysgerminomas, embryonal carcinomas, mature teratomas with malignant transformation and mixed ovarian germ cell tumors at both the transcription and translation levels. A positive correlation between KPNA2 and SALL4 expression at both the transcription level (R = 0.5120, *P* = 0.0125), and the translation level (R = 0.6636, *P*<0.0001), was presented. Extensive expression of KPNA2 was positively associated with pathologic type, recurrence and uncontrolled, ascitic fluid presence, suboptimal cytoreductive surgery necessity, resistance/refraction to initial chemotherapy, HCG level and SALL4 level in OMGCT patients. KPNA2 was found to be an independent factor for 5-year disease-free survival (DFS) of OMGCT (*P* = 0.02). The 5-year overall survival (OS) and DFS rate for KPNA2-low expression patients (88% and 79%, n = 48) were significantly higher than the OS and DFS rate for KPNA2-high expression patients (69% and 57.1%, n = 42)(*P* = 0.0151, *P* = 0.0109, respectively). The 5-year OS and DFS rate for SALL4-low expression patients (84% and 74%, n = 62) was marginally significantly higher than the high expression patients (78.6% and 71.4%, n = 28)(*P* = 0.0519, *P* = 0.0647, respectively).

**Conclusions:**

KPNA2 is a potential candidate molecular marker and important prognostic marker in OMGCT patients.

## Introduction

The ovarian malignant germ cell tumor (OMGCT) is a rare, rapid-progressing, pathologically diverse, and highly malignant form of cancer frequent in younger women. Common histological types of OMGCT include yolk sac tumors, immature teratomas, dysgerminoma and mixed ovarian germ cell tumors. Early diagnosis for OMGCT is difficult due to lacking of effective methods. The etiology and development of OMGCT remain poorly understood [1], [2]. As mammary carcinogenesis is facilitated by a sequential accumulation of genetic alterations, including the inactivation of tumor suppressor genes and the activation of oncogenes [3], the identification of specific novel genes that can be effectively used as therapeutic, diagnostic reagents and/or prognostic markers is of critical value for the treatment of OMGCT. However, ongoing research is less due to relatively small number of cases till now.

Previous reports showed ovarian germ-cell tumors and testicular germ-cell tumors to be strongly related entities that may have shared a similar origin and pathogenetic pathway [4]–[6]. Common sites of deletion were located at 3q27–q28, 5q31, 5q34–q35, 9p22–p21 and 12q22 in all histological subtypes of OGCT [7]. Immature teratomas frequently exhibit gains of chromosomes 3, 8, 12 and 14, losses of chromosomes 4 and 13, and several structural rearrangements including i(12p) [4], [8]–[12]. The most frequent anomalies for dysgerminoma were gains of 12p, 12q, 21q, 22q, 20q, 15q, 1p and 6p and the entirety of the chromosomes 19, 7, 8 and 17 [4], [9]–[11]. Amplification of MYCN was found in immature teratomas. A somatic novel missense mutation of c-KIT has been identified in one ovarian mixed dysgerminoma/EST. Recently, novel stem cell markers, SALL4, OCT4, NANOG, SOX2, UTF1, and TLC1 have been proposed as novel sensitive diagnostic markers for primary mediastinal germ cell tumors, with high specificity. Of these 6 markers, SALL4 is the only one expressed in yolk sac tumor [13]. SALL4 is also identified as a novel sensitive diagnostic marker for primary germ cell tumors of the central nervous system with high specificity, and is a more sensitive marker than α-fetoprotein and glypican-3 for yolk sac tumors [14]. A recently study evaluates the diagnostic utility of CD117, CD133, SALL4, OCT4, TCL1 and glypican-3 in OMGCT. The results exhibited OCT4 and TCL1 are useful diagnostic marker for dysgerminoma, whereas SALL4 is a more sensitive and specific marker for yolk sac tumors than glypican-3 [15]. However, the value of these markers has not been unequivocally validated.

The aim of this study was to identify potential diagnostic marker genes and molecular targets for OMGCT therapy. For this a microarray approach was first employed to select the candidate oncogenes for OMGCTs. An increased expression of KPNA2, an adaptor protein within the classic nuclear protein import machinery that mediates the import of signaling factors in the nucleus and the export of response molecules to the cytoplasm [16]–[17], was detected in OMGCTs compared to normal ovarian tissue. A series of recent reports have implicated KPNA2 expression's link to human tumorigenesis [18] such as breast cancer [19]–[24]. Our previous study also indicated that overexpression of KPNA2 in epithelial ovarian cancer correlated with a poor prognosis of patients [25]. However, it remains unclear if KPNA2 expression is related to the etiology and development of OMGCT. Based on our novel findings and said reports, we hypothesized KPNA2 to be a candidate oncogene for OMGCTs. To examine KPNA2 mRNA expression and protein changes, reverse transcription polymerase chain reaction (RT-PCR), quantitative RT-PCR, and immunohistochemistry methods were applied. As a novel known diagnostic marker for primary germ cell tumors, SALL4 was also examined in comparison with KPNA2. Correlation between KPNA and SALL4 expression, and specified OMGCT's clinicalpathologic features was further determined and assessed.

## Materials and Methods

### Patient and specimens

This study was approved by the Ethical Committee of the Cancer Center, Sun Yat-Sen University (Guangzhou, P.R. China). A total of 14 snap-frozen normal ovarian specimens, 5 mature teratomas and 23 OMGCT specimens (including 6 yolk sac tumors, 9 immature teratomas, 3 dysgerminomas, 4 mixed ovarian germ cell tumors and 1 embryonal carcinoma) collected between 2002 and 2008 were included for microarray experiment, RT-PCR and qRT-PCR. 90 OMGCT patients (including 20 yolk sac tumors, 38 immature teratomas, 11 dysgerminomas, 3 embryonal carcinomas, 10 mixed germ cell tumors, 8 mature teratomas with malignant transformation) hospitalized between 2000 and 2008 were included in this study for immunohistochemistry analysis. 90 Patients diagnosed with OMGCT at the Sun Yat-Sen University Cancer Center were treated with oophorosalpingectomy, or surgical debulking, prior to administration of chemotherapy. OMGCT tissues were dissected from the resected tumors, and normal ovarian specimens were obtained from apparently normal ovary tissue removed from other cervical carcinoma patients, after a pathological confirmation review. All samples were obtained from the Tissue Bank of Cancer Center, Sun Yat-Sen University. OMGCT specimens were evaluated by an experienced pathologist and were staged according to the Federation of Gynecology and Obstetrics (FIGO 1994) classification guidelines. Grading and histopathology subtyping of OMGCT specimens was based on criteria of the World Health Organization (WHO).

### Microarray experiment

Total RNA was extracted from 0.05 g of each snap-frozen tissue (including 3 snap-frozen normal ovarian specimens, 2 yolk sac tumors and 2 immature teratomas) using Trizol reagent (Invitrogen Life Technologies, Ontario, Canada). The samples were assessed by ethidium bromide staining before being engaged in a cDNA reverse transcription. cRNA probes were generated using the TrueLabeling-AMP™ Linear RNA Amplification Kit, and were hybridized in accordance with the manufacturer's instructions (SuperArray Bioscience, MD, USA) with purified biotin-labeled cRNA and the OHS-802 Oligo GEArray(R) Human Cancer Microarray containing 440 human oncogenes. The web-based GEArray Expression Analysis Suite program (www.sabiosciences.com) was employed for subsequent data analysis.

### RT-PCR analysis

Total RNA was extracted as described above. An RT-PCR was performed using the PrimeScript™ RT-PCR Kit (TaKaRa Bio, Shiga, Japan) according to the manufacturer's instructions in a T-gradient Biometra PCR thermal cycler (PCR-200, CA, USA). KPNA2 primer pairs included: 5′-CAAGGCTGTGGTAGATGG-3′ (forward), 5′-GCGGCAAAGATTAGAAAG-3′ (reverse) and β-actin (control) primer pairs 5′-AATCCCATCACCATCTTCCA-3′ (forward), 5′-CCTGCTTCACCACCTTCTTG-3′ (reverse). PCR products were detected through ethidium bromide staining and were semi-quantified using the Quantity One 1-D Analysis Software.

### RNA extraction and qRT-PCR analysis

To evaluate its expression level, SALL4 was also examined in comparison with KPNA2. Total RNA extraction was performed with a phenol–chloroform method, using Trizol Reagent (invitrogen, USA) for tissue lysis. The total RNA concentration and quantity were assessed by absorbency at 260 nm using a Nanodrop spectrophotometer (ND-1000, Thermo Scientific, USA). The first-strand cDNA synthesis was performed using 2 ug of total RNA and M-MLV reverse transcriptase according to the manufacturer's instructions (Promega, USA).

Real-time PCR primers and probes for KPNA2 were obtained from the Applied Biosystems Inc, CA, USA. KPNA2 primers included: 5′-CTGGGACATCAGAACAAACCAAG-3′ (forward) and 5′- ACACTGAGCCATCACCTGCAAT -3′ (reverse), SALL4 primers included: 5′- GAGTATCAGAGCCGAAGCCC-3′ (forward) and 5′- AGACTGCTCCGACCTTCCAT -3′ (reverse), and *GAPDH* (positive control) primers included: 5′- CTCCTCCTGTTCGACAGTCAGC -3′ (forward) and 5′- CCCAATACGACCAAATCCGTT -3′ (reverse). A qRT-PCR was carried out in accordance with the Platinum SYBR Green qPCR SuperMix-UDG reagents (Invitrogen, Carlsbad, CA, USA) Protocol in an ABI PRISM7700 sequence detection system (Applied Biosystems Inc, CA, USA). The ABI PRISM 7700 Cycler software was used to calculate a threshold cycle number (Ct) value for *GAPDH* and KPNA2 during the log phase of each cycle. KPNA2 levels were normalized to *GAPDH* (ΔCt = Ct_KPNA2_−Ct_GAPDH_) and were compared with the values obtained from a test sample used as a positive control through the following formula: 2^−ΔΔct^, where ΔΔCt = ΔCt_unknown_−ΔCt_positive control_. To minimize experimental variability, each sample was tested in triplicate and the mean femtogram expression level was taken as result.

### Immunohistochemistry (IHC)

Paraffin embedded tissues were analyzed using immunohistochemical staining as described by Zheng et al [25] in which the anti-KPNA2 antibody was a rabbit polyclonal antibody (1∶400) (ab84440, Abcam plc, Cambridge, UK), and anti-SALL4 antibody was a rabbit polyclonal antibody (1∶2000)(ab29112; Abcam plc, Cambridge, UK) in comparison to KPNA2. Control samples were stained in parallel, but were not incubated with either primary or secondary antibodies. Scores were determined by combining the proportion of positively stained tumor cells and the intensity of staining. Tumor cell proportions were scored as follows: 0 (no positive tumor cells); 1 (10–25% positive tumor cells); 2 (26–50% positive tumor cells); 3 (51–75% positive tumor cells) and 4 (>75% positive tumor cells). Staining intensity was graded according to the following standard: 0 (no staining); 1 (weak staining = light yellow); 2 (moderate staining = yellow brown) and 3 (strong staining = brown). The staining index (SI) was calculated as the product of the staining intensity score and the proportion of positive tumor cells. Using this method of assessment, we evaluated KPNA2 expression in normal ovarian tissues and various OMGCTs by determining the SI, with scores of 0, 1, 2, 3, 4, 6, 8, 9, or 12. Table 1, 2 tabulates the number and distribution of cases according to the immunohistochemistry score. For KPNA2, mean score is 3.73, median value 2. For SALL4, mean score is 3.26, median value 2. The cutoff value for high and low expression was determined on the basis of a measure of heterogeneity with the log-rank test statistical analysis with respect to overall survival. For KPNA2, an optimal cutoff value was determined: an SI score ≥2.5 defined tumors with high KPNA2 expression, and an SI score ≤2.5 indicated low expression. For SALL4, an optimal cutoff value was determined: an SI score ≥5 defined tumors with high SALL4 expression, and an SI score ≤5 indicated low expression. All results were confirmed by 2 or more pathologists in a double-blind analysis.

**Table 1:** Immunohistochemistry score distribution of all cases (KPNA2).

IHC score	Number of cases (%)						
	YST	IT	DYS	EC	MT	MTWMT	Total
0	2	27	2	2	2	4	39
1	0	2	1	1	1	0	5
2	2	1	1	0	2	2	8
3	2	1	2	0	0	1	6
4	1	2	0	0	1	0	4
6	4	4	1	0	1	1	11
8	2	0	2	0	0	0	4
9	3	0	0	0	1	0	4
12	4	1	2	0	2	0	9
**Total**	20	38	11	3	10	8	90

**Cutoff value:** 2.5. An SI score ≥2.5 defined tumors with high KPNA2 expression, and an SI score ≤2.5 indicated low expression. **YST:** yolk sac tumor; **IT:** immature teratoma; **DYS:** dysgerminoma; **EC:** embryonal carcinoma; **MT:** mixed germ cell tumors; **MTWMT:** mature teratomas with malignant transformation.

**Table 2:** Immunohistochemistry score distribution of all cases (SALL4).

IHC score	Number of cases (%)						
	YST	IT	DYS	EC	MT	MTWMT	Total
0	1	25	2	0	4	2	34
1	2	3	0	0	1	1	7
2	4	2	0	1	0	0	7
3	0	0	0	1	0	1	2
4	2	4	1	0	0	0	7
6	3	3	1	1	1	4	13
8	2	0	1	0	0	0	3
9	3	1	1	0	3	0	8
12	3	0	5	0	1	0	9
**Total**	20	38	11	3	10	8	90

### Statistical analysis

Fisher's exact test was used to compare the differences of KPNA2 and SALL4 expression between OMGCT and normal ovarian specimens at the levels of transcription and translation. A two-sided Fisher's exact test was also applied to study the relationship between KPNA2 expression and the histological type, histological grade, clinical stage, recurrence, patient age, and optimal cytoreduction of OMGCT performed. Survival curves for both KPNA2- low expression and KPNA2- high expression patients (as well as SALL4- high expression and SALL4- low expression patients) were plotted using the Kaplan-Meier method and were analyzed for statistical differences using log-rank tests (version5, GraphPad Software). Multivariable survival analysis using Cox's regression model was performed. Receiver operating characteristic (ROC) curve analysis was employed to determine specificity and sensitivity of KPNA2 and SALL4 expression as diagnostic tool for OMGCT. A *P* value less than 0.05 was considered statistically significant. All statistical analyses were performed using SPSS 17.0.

## Results

### Differences in gene expression for OMGCT versus normal ovarian tissues

**Figure 1:**
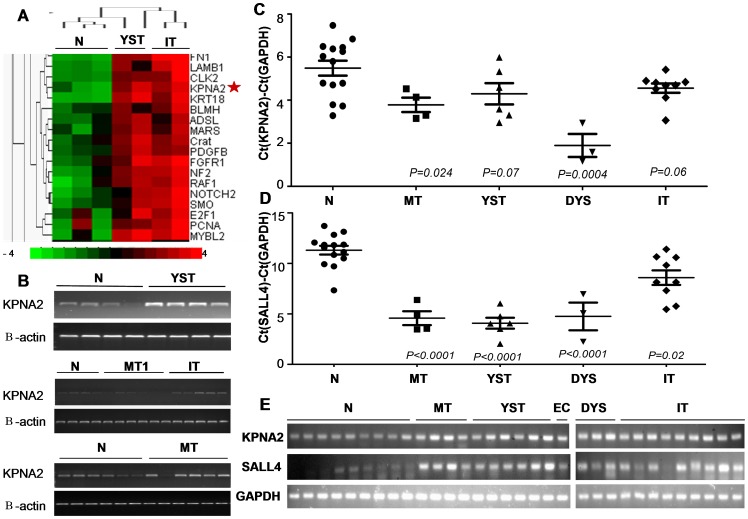
KPNA2 and SALL4 expression in OMGCT at the transcription level. **A.** Oncogene microarray assay KPNA2 is more than 8-fold overexpressed in yolk sac tumor and immature teratoma tissues compared to normal ovarian tissue. **B.** RT-PCR showed that KPNA2 expression in 3 different histological types of OMGCT, was higher than normal ovarian tissues and mature teratomas. **C,D,E.** QRT-PCR showed that both KPNA2 and SALL4 expression in 4 different histological types of OMGCT were significantly higher than normal ovarian tissue. **N**: normal ovarian tissue. **MT1**: mature teratoma. **YST**: yolk sac tumor. **IT**: immature teratoma. **MT**: mixed ovarian germ cell tumor. **DYS**: dysgerminoma.

43 genes showed overexpression in >75% of 4 OMGCT samples (2 yolk sac tumors and 2 immature teratomas) compared to 3 normal ovarian specimens, with KPNA2 exhibiting a more than 8-fold (average) comparative overexpression. KPNA2 was down-expressed in the normal ovarian specimens but extensively overexpressed in 2 yolk sac tumors and 2 immature teratomas (Figure 1A).

### Transcription and translation of KPNA2 and SALL4 in OMGCT versus normal ovarian tissues

RT-PCR showed that KPNA2 expression in 3 different histological types of OMGCT(including 4 yolk sac tumors, 5 immature teratomas, and 6 mixed ovarian germ cell tumors), was higher than normal ovarian tissues and mature teratomas (Figure 1B).

QRT-PCR results demonstrate a higher KPNA2 expression in all types of OMGCT (including dysgerminomas, yolk sac tumors, immature teratomas, mixed ovarian germ cell tumors and embryonal carcinoma) in comparison with normal ovarian tissues (1.46±1.36,mean±SD), with KPNA2 over-expression in mixed ovarian germ cell tumors (3.89±1.44, mean±SD), yolk sac tumors (3.09±1.70, mean±SD), dysgerminomas (6.04±5.95, mean±SD) and immature teratomas (5.14±5.64, mean±SD) being the most significant (*P* = 0.024, *P* = 0.07, *P* = 0.0004, *P* = 0.06, respectively) (Figure 1C). Extensive KPNA2 expression was observed in 3/3 dysgerminomas, 4/6 yolk sac tumors, 7/9 immature teratomas and 4/4 mixed ovarian germ cell tumors (mixed with yolk sac tumors and embryonal carcinomas), and 1/1 embryonal carcinoma. QRT-PCR assay determined the expression level of KPNA2 in dysgerminomas, yolk sac tumors, mixed ovarian germ cell tumors, and immature teratoma as 4.13 times, 2.11 times, 2.66 times and 3.5 times higher pronounced than normal ovarian specimens, respectively (Figure 1C,E).

A similar distribution was observed in all types of OMGCT for SALL4. SALL4 expression in mixed ovarian germ cell tumors (90.44±44.53, mean±SD), yolk sac tumors (64.45±37.55, mean±SD), dysgerminomas (43.56±25.14, mean±SD) and immature teratomas (24.79±23.80, mean±SD) was significantly higher than normal ovarian tissues (*P*<0.0001, *P*<0.0001, *P*<0.0001, *P* = 0.02, respectively) (Figure 1D). Extensive SALL4 expression was observed in 3/3 dysgerminomas, 6/6 yolk sac tumors, 6/9 immature teratomas and 4/4 mixed ovarian germ cell tumors (mixed with yolk sac tumors and embryonal carcinomas), 1/1 embryonal carcinoma, compared with the normal ovarian specimens. QRT-PCR assay showed the expression level of SALL4 in dysgerminomas, yolk sac tumors, mixed ovarian germ cell tumors, and immature teratoma to be pronounced 20.39 times, 30.17 times, 42.34 times and 11.6 times higher than normal ovarian specimens, successively (Figure 1D,E).

**Figure 2:**
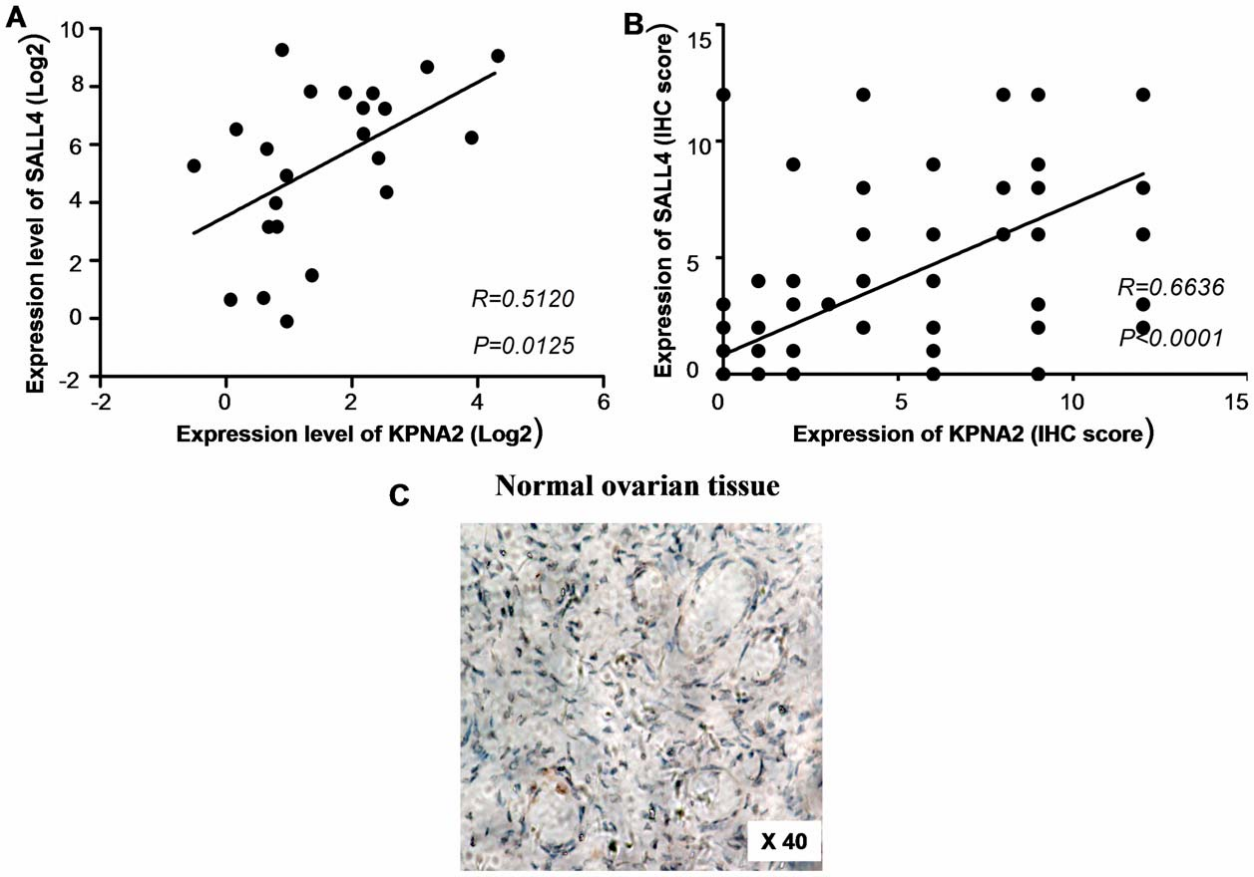
Correlation between KPNA2 expression and SALL4 expression. **A.** A positive correlation between KPNA2 and SALL4 expression was observed at the transcription level. **B.** A positive correlation between KPNA2 and SALL4 expression was observed at the translation level. **C.** Negative IHC staining was observed in normal ovarian tissue. (Ori. mag. ×40).

**Figure 3:**
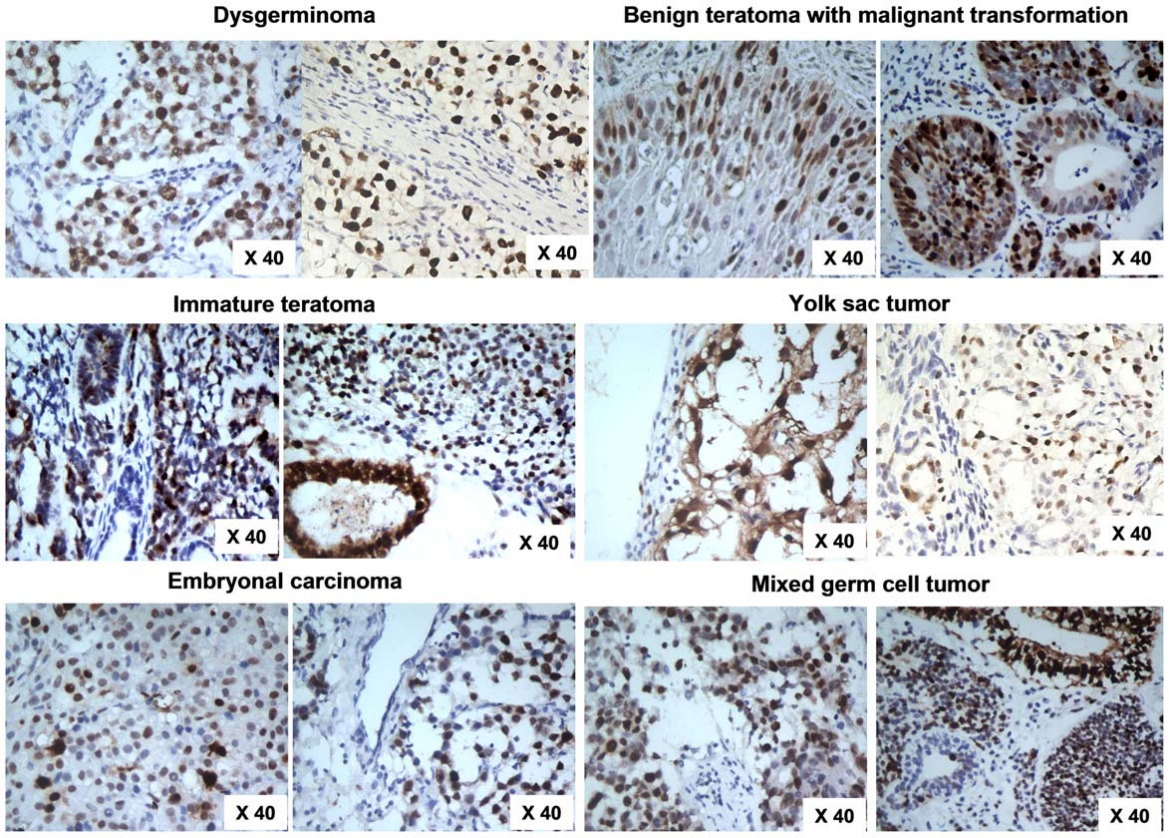
KPNA2 and SALL4 expression in OMGCT at the translation level. Positive IHC staining in dysgerminoma, benign teratoma with malignant transformation, immature teratoma, yolk sac tumor, embryonal carcinoma, and mixed germ cell tumor (Ori. mag. ×40).

**Figure 4:**
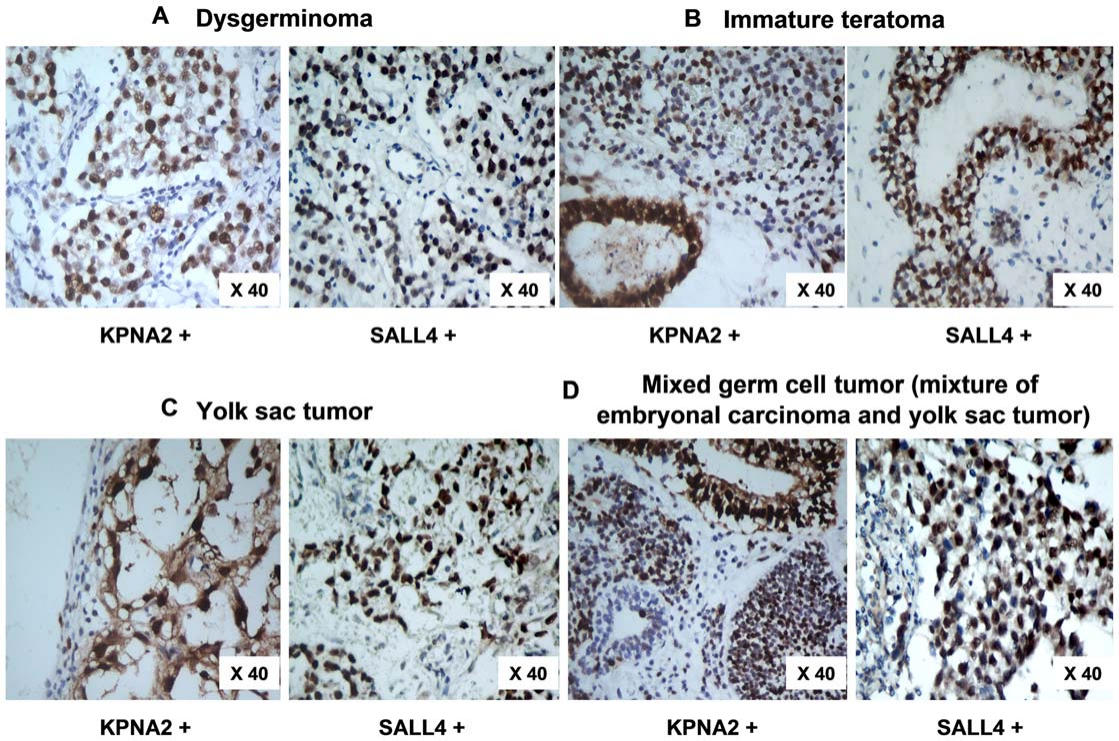
Comparison between KPNA2 and SALL4 expression in OMGCT. Comparison between KPNA2 and SALL4 expression in dysgerminoma, immature teratoma, yolk sac tumor, and mixed germ cell tumor (Ori. mag. ×40).

Immunocytochemistry analysis revealed that both the KPNA2 and SALL4 protein were located primarily in the cell nucleus. At the translation level, negative KPNA2 expression (KPNA2 −/+) and SALL4 (SALL4 −/+) was observed in all normal ovarian specimens (Figure 2C). In contrast, cell nuclear intensely labeled with KPNA2 were present in 65% (13/20) of yolk sac tumors, 26.7% (8/38) of immature teratomas, 81.8% (9/11) dysgerminomas, 66.7% (2/3) embryonal carcinomas, and 50% (5/10) mixed ovarian germ cell tumors (mixed with yolk sac tumors and embryonal carcinomas) (Figure 3, Table 1), while those labeled with SALL4 were present in 65% (13/20) of yolk sac tumors, 13.2% (5/38) of immature teratomas, 45.5% (5/11) dysgerminomas, 0% (0/3) embryonal carcinomas, and 40% (4/10) mixed ovarian germ cell tumors (mixed with yolk sac tumors and embryonal carcinomas) (Figure 4, Table 2).

### Correlation between KPNA2 expression and SALL4 expression at the transcription and the translation levels

Correlation between KPNA2 expression and SALL4 expression was analyzed. Results indicated a positive correlation between KPNA2 and SALL4 expression (R = 0.5120, *P* = 0.0125, respectively) at the transcription level (Figure 2A), and a positive correlation between KPNA2 and SALL4 expression (R = 0.6636, *P*<0.0001, respectively) at the translation level (Figure 2B).

### Correlation between KPNA2 expression and clinicopathologic features of OMGCT

Table 3 lists the clinicopathologic features of 90 OMGCT patients from whom specimens were taken for KPNA2 expression analysis in this study. Higher expression of KPNA2 was significantly associated with pathologic type (*P* = 0.002), recurrence and uncontrolled (*P* = 0.039), ascitic fluid presence (*P* = 0.037), suboptimal cytoreductive surgery necessity (*P* = 0.008), resistance/refraction to initial chemotherapy (*P* = 0.037), HCG level (*P* = 0.022) and SALL4 level (*P* = 0) in 90 OMGCT patients (Table 3).

**Table 3:** Correlations between KPNA2 expression and clinicopathological features of patients with OMGCT.

	OMGCT			Immature teratoma		
	KPNA2			KPNA2		
Characteristics	Low(n = 48)	High(n = 42)	*P* * *value*	Low(n = 30)	High(n = 8)	*P* * *value*
**Pathologic type**						
Yolk sac tumor	7	13				
Immature teratoma	30	8				
Dysgerminoma	2	9				
Embryonal carcinoma	1	2				
Mixed germ cell tumor	5	5				
Mature teratoma with malignant transformation	3	5	**0.002**			
**Tumor size**						
<19 cm	27	25		16	4	
≥19 cm	21	17	0.832	14	4	1
**Tumor grade**						
G1				11	0	
G2				13	3	
G3				6	5	**0.031**
**FIGO stage**						
I	25	19		13	0	
II	6	6		11	3	
III–IV	17	17	0.811	6	5	**0.025**
**Recurrence and uncontrolled**						
No	38	24		25	3	
Yes	10	18	**0.039**	5	5	**0.019**
**Ascitic fluid**						
Without	28	15		18	3	
With	20	27	**0.037**	12	5	0.426
**Cytoreductive surgery**						
Suboptimal	0	6				
Optimal	48	36	**0.008**	30	8	1
**Ascitic fluid cytology**						
Negative	47	38		29	8	
Positive	1	4	0.181	1	0	1
**CEA**						
<5 µg/ml	23	17		11	4	
≥5 µg/ml	5	3		2	0	
Missing data	20	12	0.703	17	4	1.0
**Response to initial chemotherapy**						
Sensitive	35	24		22	3	
Resistant or refractory	6	14		3	5	
Missing data	7	4	**0.037**	5	0	**0.01**
**AFP**						
<25 µg/ml	20	17		18	4	
≥25 µg/ml	20	18		9	4	
Missing data	8	7	1	3	0	0.433
**HCG**						
<3 µg/ml	21	17		23	5	
≥3 µg/ml	2	11		2	1	
Missing data	25	14	**0.022**	5	2	0.488
**SALL4**						
Low	46	16		30	2	
High	2	11	**0**	0	6	**0**

* **Two-sided Fisher's exact test**.

**G1**: Well differentiated (Low grade); **G2**: Moderately differentiated (Intermediate grade); **G3**: Poor differentiated (High grade).

### Correlation between KPNA2 expression and clinicopathologic features of immature teratoma patients

Furthermore, high expression of KPNA2 expressed significant association with a poor tumor grade (*P* = 0.031), an advanced FIGO stage (*P* = 0.025), recurrence and uncontrolled (*P* = 0.019), resistance/refraction to initial chemotherapy (*P* = 0.01), and SALL4 level (*P* = 0) in 38 immature teratoma patients (Table 3).

### Uni- and Multi-variable analysis of clinicopathologic factors associated with 5-year OS and DFS rates of OMGCT patients

Patient follow-up data was available for all 90 individuals of OMGCT. Duration of follow-up ranged from 2.23–160.63 months, with a median follow-up period of 88.27 months, and by the time of last contact 19 total deaths had ocurred due to persistent or recurrent local tumors and distant metastases. The 5-year overall survival (OS) rate was 82%(74/90) for all cases, including 80%(16/20) for the 20 yolk sac tumors, 89%(34/38) for the immature teratomas, 100% (11/11) for the dysgerminomas, 67.7% (2/3) for the embryonal carcinomas, and 60% (6/10) for the mixed ovarian germ cell tumors. The 5-year disease-free survival (DFS) rate was 76%(29/90) for the OMGCT patients, including 70%(14/20) for the yolk sac tumors, 76%(29/38) for the immature teratomas, 91% (10/11) for the dysgerminomas, 66.7% (2/3) for the embryonal carcinomas, and 50% (5/10) for the mixed ovarian germ cell tumors.

Univariable analysis using log-rank tests showed that KPNA2 expression (*P* = 0.007), stage (*P* = 0.011), cytoreductive surgery (*P* = 0.019), and response to initial chemotherapy (*P* = 0) as factors significantly associated with the 5-year DFS rate for 90 OMGCT patients. Multivariable analysis showed that KPNA2 expression (*P* = 0.02) and response to initial chemotherapy (*P* = 0) were factors significantly associated with the 5-year DFS rate for these patients. Both analyses determined SALL4 expression as being insignificant in affecting the 5-year DFS rate (Table 4).

**Table 4:** Cox regression analysis of the various factors associated with disease-free survival and overall survival in OMGCT patients.

Disease-free survival		IHC data sets	
Variables	Favorable/Unfavorable	HR(95% CI)	*P value*
**Univariables**			
KPNA2	high/low	3.192 (1.433–7.11)	**0.007**
FIGO Stage	III–IV/I–II	2.804 (1.269–6.195)	**0.011**
Cytoreductive surgery	optimal/suboptimal	3.251 (1.214–8.707)	**0.019**
Response to initial chemotherapy	resistant or refractory/sensitive	9.86 (4.288–22.676)	**0**
SALL4	high/low	4.105 (1.944–12.696)	0.069
**Multivariate analysis**			
KPNA2	high/low	2.014 (1.251–12.882)	**0.02**
FIGO Stage	III–IV/I–II	0.809 (0.308–2.124)	0.667
Cytoreductive surgery	suboptimal/optimal	3.167 (0.974–10.292)	0.055
Response to initial chemotherapy	resistant or refractory/sensitive	8.855 (4.568–26.178)	**0**
SALL4	high/low	3.312 (1.85–7.146)	0.079

Overall survival			
Variables	Favorable/Unfavorable	HR(95% CI)	*P*
**Univariables**			
KPNA2	high/low	3.388(1.203–9.54)	**0.021**
FIGO Stage	III–IV/I–II	4.556(1.604–12.944)	**0.004**
Cytoreductive surgery	optimal/suboptimal	3.492(0.991–12.301)	0.052
Response to initial chemotherapy	resistant or refractory/sensitive	6.278(5.739–19.438)	**0**
SALL4	high/low	3.477(1.362–8.874)	**0.039**
**Multivariate analysis**			
KPNA2	high/low	2.4(1.43–8.352)	0.106
FIGO Stage	III–IV/I–II	1.672(0.46–6.08)	0.435
Cytoreductive surgery	optimal/suboptimal	1.714(0.333–8.812)	0.519
SALL4	high/low	2.13(0.592–7.667)	0.147
Response to initial chemotherapy	resistant or refractory/sensitive	7.431(5.989–25.638)	**0.002**

**HR**, Harzard radio; **CI**, Confidence interval.

In addition, univariable analysis showed that KPNA2 expression (*P* = 0.021), stage (*P* = 0.004), response to initial chemotherapy (*P* = 0) and SALL4 level (*P* = 0.039) were factors significantly associated with the 5-year OS rate for patients. Multivariable analysis expressed patients' response to initial chemotherapy (*P* = 0.002) as the sole factor significantly associated with the 5-year OS for 90 OMGCT patients (Table 4).

### Prognostic assessment of positive KPNA2 and SALL4 expression in 90 OMGCT patients

**Figure 5:**
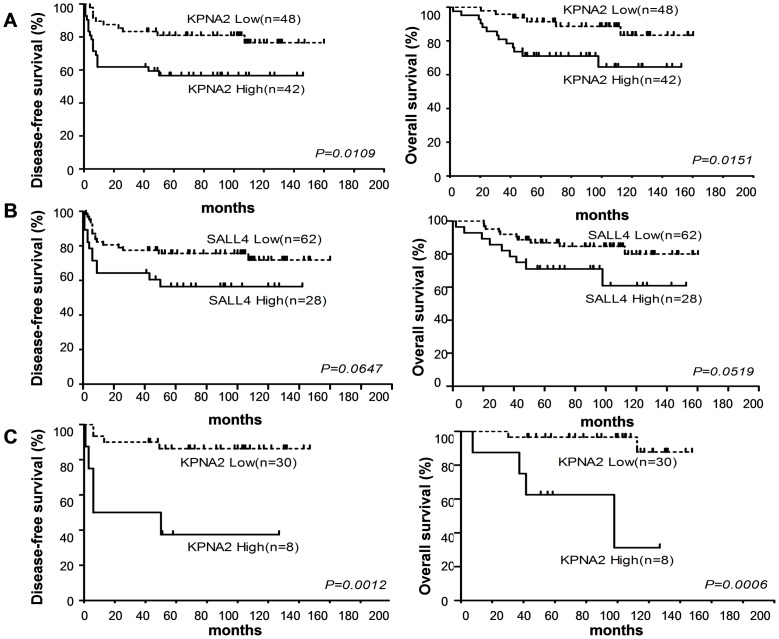
Prognostic assessment of positive KPNA2 and SALL4 expression in OMGCT and immature teratoma patients. **A.** There was statistically significant difference in both 5-year OS rate and DFS between KPNA2-low and KPNA2-high expression patients. **B.** There was marginally significant difference in both 5-year OS rate and DFS between SALL4-low and SALL4-high expression patients. **C.** There was significant difference in both 5-year OS rate and DFS between KPNA2-low and KPNA2-high expression immature teratoma patients. The graph plotted between estimated survival probabilities/estimated survival percentages (on Y axis) and time past after entry into the study (on X axis) consists of horizontal and vertical lines.

Kaplan-Meier survival curves show an inverse correlation between both KPNA2 and SALL4 expression and patient survival rate (Figure 5A, 5B). Using the log-rank test, the 5-year OS rate for KPNA2- low expression patients (88%, n = 48) was calculated to be significantly higher than the OS rate for KPNA2-high expression patients (69%, n = 42)(*P* = 0.0151). The 5-year DFS rate for KPNA2- low expression patients (79%, n = 48) was also significantly higher than the DFS rate for high expression patients (57.1%, n = 42)(*P* = 0.0109).

However, the 5-year OS rate for SALL4-low expression patients (84%, n = 62) was calculated to be marginally significantly higher than the high expression patient group (78.6%, n = 28)(*P* = 0.0519). The 5-year DFS rate for SALL4-low expression patients (74%, n = 62) was also marginally significantly higher than the DFS rate for SALL4-high expression patients (71.4%, n = 28)(*P* = 0.0647).

### Prognostic assessment of positive KPNA2 expression in 38 immature teratoma patients

Moreover, kaplan-Meier survival curves show that immature teratoma patients exhibiting higher KPNA2 expression had substantially lower survival rates than the patients who had lower KPNA2 expression (Figure 5C). The 5-year OS rate for KPNA2-low expression patients (93%, n = 30) was calculated to be significantly higher than the OS rate for KPNA2-high expression patients (50%, n = 8)(*P* = 0.0012). The 5-year DFS rate for KPNA2-low expression patients (87%, n = 30) was also significantly higher than the DFS rate for KPNA2-high expression patients (37.5%, n = 8)(*P* = 0.0006).

## Discussion

Our research presents an exciting novel finding on KPNA2 expression associating with OMGCT. In this study, we described the identification and characterization of KPNA2 expression in several common pathological types of OMGCTs, including 20 yolk sac tumors, 38 immature teratomas, 11 dysgerminomas, 3 embryonal carcinomas, 10 mixed germ cell tumors, 8 mature teratomas with malignant transformation, at both transcription and translation levels. Additionally we demonstrated a correlation between KPNA2 expression and the OMGCT's pathologic types, recurrence and uncontrolled, ascitic fluid presence, suboptimal cytoreductive surgery necessity, resistance/refraction to initial chemotherapy, HCG level and SALL4 level. KPNA2 was proven an unfavorable prognostic indicator for the OS and DFS of OMGCT patients.

In this study, we first utilized the Oligonucleotide Cancer Microarray to identify candidate oncogenes of OMGCTs, of which 43 were determined (Data omitted due to study focus on KPNA2). KPNA2, due to its extensive expression in OMGCTs, was identified as a candidate gene. KPNA2 was down-expressed in normal ovarian tissue but extensively over-expressed in 2 yolk sac tumors and 2 immature teratoma. Such findings provide valuable insight into the critical role of the KPNA2 gene in yolk sac tumor and immature teratoma histopathologic types of OMGCT tumorigenesis.

KPNA2 (also named RAG cohort 1 and importin alpha 1), a human gene located in 17q24.2, is an adaptor protein within the classic nuclear protein import machinery; such mediates the import of signaling factors in the nucleus and the export of response molecules to the cytoplasm [16], [26]. KPNA2 has been known to play an important role in regulating epidermal proliferation and differentiation, and activating cellular signaling in blood lymphocytes [17], [27]. KPNA2 has also been associated with the Russell-Silver syndrome [28] and possess tumorigenic activity [18], [29]. A series of reports suggest that KPNA2 expression is linked to breast carcinogenesis and acts as a potential prognostic marker for breast carcinomas [20]–[23]. Thakur reported that BRCA1 is a protein transported into the nucleus through the karyopherin pathway [19], possibly associating KPNA2 expression with breast carcinogenesis. New research showed that forced KPNA2 expression drives malignant features relevant to breast cancer progression, while its silencing is required for the remission of those progressive phenotypes. This study gives clear evidence that KPNA2 acts as a novel oncogenic factor in human breast cancer, in vitro [30]. Numerous studies demonstrate that mutations in BRCA1 or BRCA2 can predispose one to breast and ovarian cancer, among other cancers [31]–[35]. Women who carry BRCA1 or BRCA2 genetic mutations appear to retain a high lifetime risk of developing ovarian cancer. Furthermore, recent work identified KPNA2 as a potential prognostic marker for the progression of prostate cancer [36], non-muscle-invasive bladder cancer [37], as well as non-small cell lung cancer [38], hepatocellular carcinoma. Our previous study has demonstrated that overexpression of KPNA2 is associated with the prognosis of epithelial ovarian cancer [25]. Thus, basing upon our microarray finding of KPNA2 overexpression in OMGCTs and report evidence, we predicted that KPNA2 might also reveal potential new targets for OMGCTs. Before now, there has been no study conclusively evidencing KPNA2 expression as being associated with OMGCTs.

We subsequently confirmed the findings from microarrays at both transcription and translation levels and further explored whether the activity of KPNA2 could be correlated with specific clinicopathological characteristics. QRT-PCR data supported the observation of extensive KPNA2 expression in several common pathological types of OMGCTs including yolk sac tumors, immature teratomas, dysgerminomas, mixed ovarian germ cell tumors and embryonal carcinoma when compared with normal ovarian tissues. Immunohistochemistry confirmed that increased KPNA2 protein expression was presented in yolk sac tumors, immature teratomas, dysgerminomas, embryonal carcinomas, and mixed ovarian germ cell tumors, whereas negative KPNA2 expression was observed in all normal ovarian specimens. Our study is the first to associate KPNA2 expression with human OMGCTs development, other than human breast cancer as cited above.

To expand our vision and find more convincing proof, SALL4 expression was performed in parallel in qRT-PCR and immunohistochemistry for comparison with KPNA2. SALL4 belongs to a family of zinc finger transcription factors and is a novel finding stem cell biomarker which has been identified for germ cell tumors of central nervous system and primary mediastinum. It has higher specificity and is more sensitive than α-fetoprotein and glypican-3 for yolk sac tumor types [13], [14]. A similar finding was also presented in OMGCT recently [15]. Our study demonstrates that KPNA2 tends to be expressed less than SALL4 at both the mRNA and protein level in OMGCT, in comparison to normal ovarian tissue. Furthermore, a positive correlation between KPNA2 and SALL4 expression was observed at both transcription level (R = 0.5120, *P* = 0.0125) and translation level (R = 0.6636, *P*<0.0001) in OMGCT. The interaction mechanism between them is worthy of further exploration.

Based on our above immunohistochemistry findings, we further explored the clinical significance of KPNA2 and SALL4 expression in OMGCT. Our results indicated KPNA2 to be a novel independent prognostic marker for disease progression in OMGCT, whereas SALL4 was insignificant in affecting the 5-year DFS and OS of OMGCT patients. KPNA2's extensive expression was also found to be significantly associated with pathologic type, recurrence and uncontrolled, ascitic fluid presence, suboptimal cytoreductive surgery necessity, resistance/refraction to initial chemotherapy, HCG level and SALL4 level in 90 OMGCT patients. KPNA2-extensive expression patients have also a significantly lower OS and DFS when compared to KPNA2-low expression patients (*P* = 0.0151, *P* = 0.0109, respectively). It is most likely that KPNA2 plays an important role in the development, differentiation, and carcinogenesis of OMGCT. These results provided further evidence and indication that KPNA2 might be recognized as an important diagnostic marker for OMGCT patients.

Several studies indicated that KPNA2 extensive expression in breast cancer patients was associated with a short overall patient survival and recurrence-free survival [20], [28], and may warrant consideration as a potential marker for chemoresistance in advanced breast cancer [24]. Our study indicated that KPNA2 extensive expression strongly associates with poor prognosis and recurrence rates of OMGCT patients, as consistent with previous reports. Although SALL4 is a useful diagnostic marker for primary mediastinal germ cell tumors [13], primary germ cell tumors of the central nervous system [14], yolk sac tumors [15], *et al*, however, there is no study so far on the role of SALL3 in prognosis of OMGCT. Our study presented that SALL4 might be a possible prognosis mark for OMGCT.

In all OMGCT disease, 38 cases were immature teratomas, thus leading to a focus study in this area. Immunohistochemistry results indicated that high expression of KPNA2 expressed significant association with a poor tumor grade, an advanced FIGO stage, recurrence and uncontrolled, resistance/refraction to initial chemotherapy, and SALL4 level in these patients. Similar to various OMGCTs, KPNA2-extensive expression immature teratomas patients have a significantly lower OS and DFS when compared to KPNA2-negative patients (*P* = 0.0012, *P* = 0.0006, respectively). This finding is consistent with the above results.

In conclusion, our study demonstrates that extensive expression of KPNA2 is present, and is positively associated with pathologic type, recurrence and uncontrolled, ascitic fluid presence, suboptimal cytoreductive surgery necessity, resistance/refraction to initial chemotherapy, HCG level and SALL4 level in OMGCT patients. KPNA2 extensive expression links to a poor prognosis and disease progression for OMGCT patients. SALL4 also distributes positive expression in OMGCT, with its expression marginally correlating with a poor prognosis and disease progression. A positive correlation also exists between KPNA2 and SALL4 expression in OMGCT. Thus, our study provides evidence that KPNA2 might play a critical role in the development, differentiation and carcinogenesis of OMGCT, and may be utilized as an important diagnostic marker and indicator of poor prognosis for OMGCT patients.
